# Non‐Invasive Assessment of Endothelial Dysfunction and Arterial Stiffness in Fontan Circulation

**DOI:** 10.1111/eci.70257

**Published:** 2026-09-15

**Authors:** Amalia Baroutidou, Eleni Pagkopoulou, Vasileios Kamperidis, Antonios Ziakas, Theodoros Dimitroulas, George Giannakoulas

**Affiliations:** ^1^ First Department of Cardiology, AHEPA University Hospital Aristotle University of Thessaloniki Thessaloniki Greece; ^2^ Fourth Department of Internal Medicine, Hippokration University Hospital, Medical School Aristotle University of Thessaloniki Thessaloniki Greece

**Keywords:** arterial stiffness, endothelial dysfunction, Fontan, single ventricle, univentricular heart, vascular dysfunction

## Abstract

**Background:**

The Fontan procedure constitutes the definitive surgical strategy for complex congenital heart disease, establishing a circulation in which systemic venous blood is directed to the pulmonary arteries through a staged reconstruction that separates systemic and pulmonary venous return in the absence of a subpulmonary ventricle. This uniquely altered haemodynamic state exerts deleterious effects on endothelial function, thereby promoting progressive end‐organ dysfunction and contributing substantially to the long‐term morbidity observed in patients with Fontan circulation.

**Μethods/Results:**

Over recent decades, clinical research has employed a range of functional methods to assess macro‐ and microvascular function, as well as arterial stiffness. This review summarizes the available functional non‐invasive techniques for evaluating vascular function in the Fontan circulation and discusses the existing evidence linking these vascular parameters to cardiac dysfunction, associated comorbidities, and clinical outcomes in this population.

**Conclusions:**

Functional non‐invasive vascular assessment may provide valuable information for monitoring disease progression and risk stratification in Fontan circulation; however, further longitudinal studies are required to clarify its prognostic significance and potential clinical utility.

## Graphical abstract

The Fontan procedure is the definitive treatment for complex congenital heart disease, creating a circulation without a subpulmonary ventricle. This altered hemodynamic state negatively affects endothelial function, contributing to progressive organ dysfunction and long‐term morbidity. Various noninvasive methods have been used to assess macro‐ and microvascular function and arterial stiffness. This review outlines these techniques and examines evidence linking vascular dysfunction to cardiac impairment, comorbidities and clinical outcomes in patients with Fontan circulation. The central illustration provides an overview of non‐invasive techniques for evaluating endothelial dysfunction and arterial stiffness in patients with Fontan circulation. FMD, Flow‐mediated dilatation; NIRS, Near‐Infrared Spectroscopy; NMD, nitroglycerin‐mediated dilation; PAT, Peripheral arterial tonometry; PWV, Pulse wave velocity; VOP, Venous occlusion plethysmography.

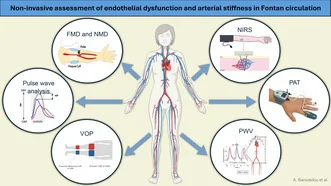

**Abbreviations**
ACEi: angiotensin‐converting enzyme inhibitors
AIx: Augmentation Index
AP: augmentation pressure
AVDO_2_
: arterial–venous oxygen saturation differences
eNOS: endothelial nitric oxide synthase
FBF: forearm blood flow
FMD: flow‐mediated dilatation
FVC: forearm vascular conductance
Hb: haemoglobin
HLHS: hypoplastic left heart syndrome
Mb: myoglobin
MRI: magnetic resonance imaging
MSNA: muscle sympathetic nerve activity
NIRS: Near‐Infrared Spectroscopy
NMD: nitroglycerin‐mediated dilation
NO: nitric oxide
PAT: peripheral arterial tonometry
PLE: protein‐losing enteropathy
PP: pulse pressure
PWA: pulse wave analysis
PWV: pulse wave velocity
RHI: Reactive Hyperemia Index
ROS: reactive oxygen species
rSO2: regional oxygen saturation
VO_2_
: oxygen consumption
VOP: venous occlusion plethysmography

## 1 Introduction

Congenital heart disease constitutes the most common congenital abnormality worldwide, affecting ~1% of newborns, whereas a rarer subset—approximately 1/3000 neonates—presents with a variety of cardiac malformations characterized by a functionally single ventricle [1, 2]. The Fontan procedure has emerged as the therapeutic approach of choice for managing these intricate cardiac defects [3]. This multistage repair facilitates the passive flow of systemic venous blood into the pulmonary circulation, effectively bypassing the subpulmonary ventricle [3]. Initially introduced in 1968 as an atrio‐pulmonary connection, the procedure has since been replaced by the total cavopulmonary connection, which employs either an intracardiac or extracardiac conduit to establish a connection between the inferior vena cava and the pulmonary artery [3].

The development of the Fontan procedure, along with the subsequent Fontan‐type modifications, has markedly improved survival outcomes in individuals with single‐ventricle physiology [3, 4]. As a result, an increasing number of these patients are now reaching adulthood, with many surviving into their fourth decade of life and beyond [4, 5]. Despite significant advancements in early outcomes, long‐term survival rates deteriorate over time, accompanied by a progressive increase in cardiovascular morbidity [3]. The hemodynamic compromise in Fontan circulation may be partially attributed to the absence of a subpulmonary ventricle, resulting in persistent systemic venous congestion, altered pulmonary hemodynamics, and a ventricle chronically deprived of adequate preload [3].

**FIGURE 1:**
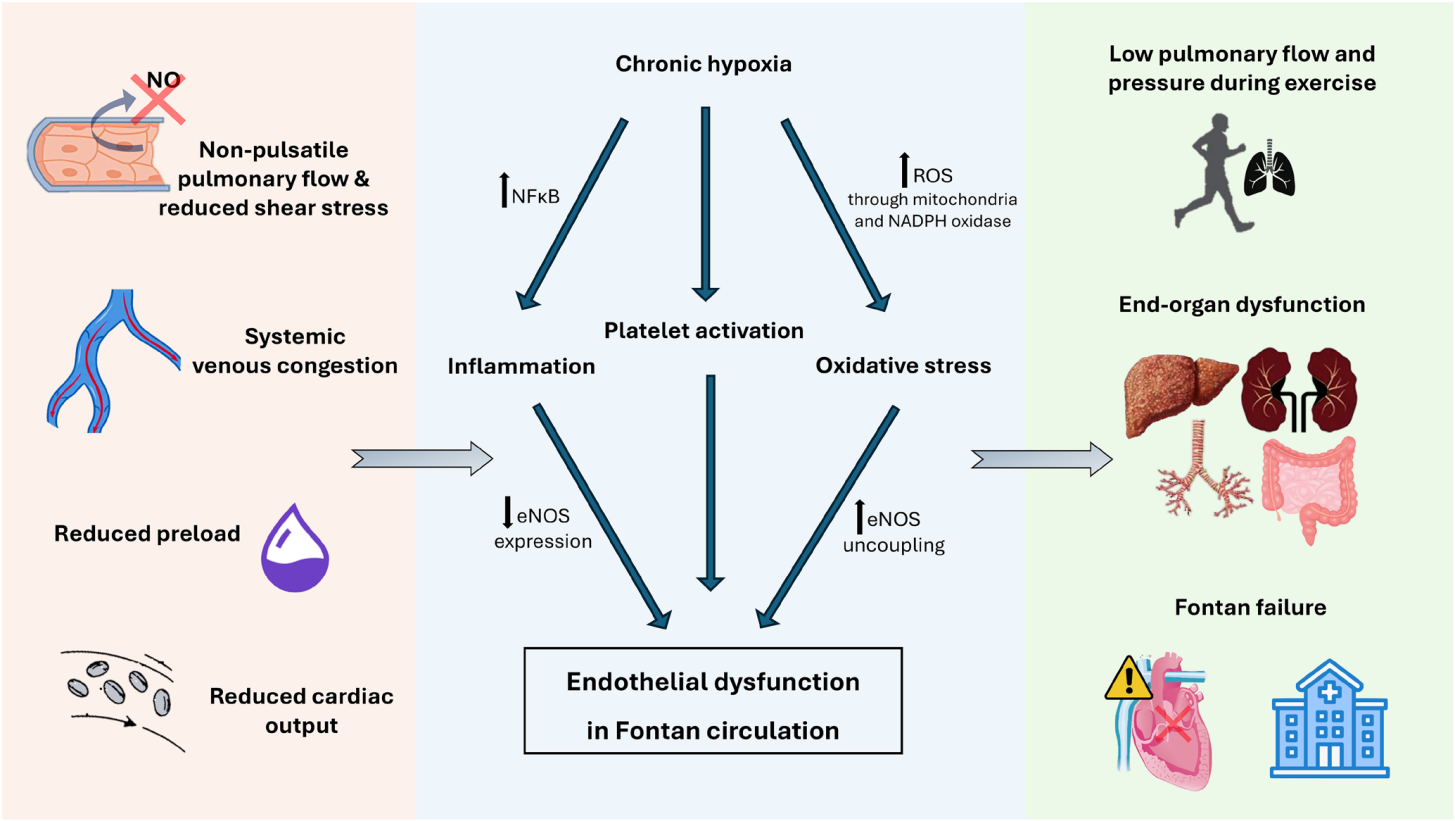
Overview of the interplay between Fontan circulation hemodynamics, endothelial dysfunction, end‐organ dysfunction, exercise intolerance and Fontan failure. eNOS, endothelial nitric oxide synthase; NADPH, nicotinamide adenine dinucleotide phosphate; NF‐κB, nuclear factor κB; NO, nitric oxide; ROS, reactive oxygen species.

The adverse consequences of Fontan physiology extend beyond the altered cardiac circulation as increasing evidence suggests that vascular dysfunction—characterized by endothelial dysfunction, impaired tissue oxygenation and increased arterial stiffness—represents an important systemic component of Fontan pathophysiology. Collectively, these vascular alterations may be regarded as a downstream systemic consequence of the absence of a subpulmonary ventricle in Fontan circulation, mechanistically linked to chronic venous congestion, impaired cardiac output, loss of pulsatile pulmonary blood flow, abnormal shear stress signalling and chronic tissue hypoxemia. Within this framework, vascular dysfunction assessment may provide valuable insights into the complex interplay between abnormal Fontan haemodynamics and the vascular compartment, possibly elucidating the pathophysiological cascade linking the Fontan circulation to long‐term cardiovascular complications and the progression to late Fontan failure [6, 7, 8, 9, 10] (Figure 1). Over the past decades, clinical research on endothelial dysfunction has employed several functional methods—originally developed for biventricular circulation—that allow the assessment of macro‐ and microangiopathy. A recent meta‐analysis demonstrated widespread endothelial injury across both the macro‐ and microvasculature in adults with congenital heart disease, assessed via semi or non‐invasive techniques [11]; however, the presence and role of endothelial dysfunction within the specific population of Fontan patients remain poorly understood.

In the light of the above, we provide a comprehensive overview of the contemporary methods for the evaluation of endothelial function and arterial stiffness in patients with Fontan circulation. Potential associations between vascular dysfunction parameters and various indicators of cardiac dysfunction, alongside related comorbid conditions, are also discussed. Finally, key challenges and future research directions in this challenging patient population are outlined.

## 2 Assessment of Endothelial Dysfunction in Fontan Circulation: Functional Methods

### 2.1 Venous Occlusion Plethysmography

Venous occlusion plethysmography (VOP) was one of the earliest techniques described for the assessment of vascular function in vivo and has been regarded for many years as the ‘gold standard’; however, it is no longer routinely used due to its semi‐invasive nature and time‐consuming procedures [12, 13]. The principle of VOP relies on measuring tissue blood flow by monitoring changes in tissue volume [13]. These volume changes are provoked by inflating a cuff around the upper portion of the limb under examination, to a pressure that exceeds venous pressure but remains below arterial pressure levels [14, 15]. This temporarily impedes venous drainage, while preserving arterial inflow, allowing blood to enter the limb but preventing its exit [13]. Subsequently, the resulting tissue volume change rate is proportional to arterial inflow rate [13, 14]. The cuff is usually inflated to approximately 40 mmHg for 10 s, followed by a 5‐s deflation period, a cycle that ensures venous emptying without affecting arterial inflow [13]. Tissue volume changes are quantified by a plethysmograph; most commonly an automatically calibrated mercury‐in‐rubber (or silastic) strain gauge is placed around the widest part of the limb [13]. Additionally, to accurately measure blood flow in the calf or forearm, an occlusive cuff is typically inflated above systolic pressure on the hand or foot [15]. This effectively excludes blood flow from the distal extremities, ensuring that the recorded measurements reflect proximal limb circulation exclusively [15].

Vascular responsiveness is assessed by measuring forearm volume changes (expressed in mL/min/100 mL) induced by reactive hyperemia or vasoactive agents' infusion [16]. Endothelium‐dependent vasodilation is evaluated through the administration of nitric oxide (NO) agonists, including acetylcholine, bradykinin, adenosine triphosphate, endothelin‐1, serotonin, histamine and thrombin. Conversely, endothelium‐independent vasodilation is examined by administering vascular smooth muscle relaxing agents, for example, nitrates [16].

Only one study has evaluated endothelial function via VOP in Fontan circulation so far, reporting blunted forearm blood flow (FBF) and forearm vascular conductance (FVC) in Fontan patients compared to controls [17]. In this study, FVC was negatively correlated with muscle sympathetic nerve activity (MSNA), suggesting that the increase of systemic vascular resistance, as a potential adaptive mechanism to chronic suboptimal cardiac output in Fontan circulation, may contribute to diminished peripheral blood flow [17].

### 2.2 Flow‐Mediated Dilatation

Over the past decades, Flow‐Mediated Dilatation (FMD) has become the most widely used non‐invasive technique for quantifying endothelial function in conduit arteries [18]. This technique measures arterial diameter (most commonly of the brachial artery) using ultrasound imaging at baseline and following reactive hyperemia, induced by releasing a blood pressure cuff previously inflated to supra‐systolic blood pressure levels for 5 min [18, 19]. Although the initial phase of reactive hyperemia following transient arterial occlusion is largely mediated by the ischemia‐induced reduction in peripheral vascular resistances and by local metabolic mechanisms, which are predominantly endothelium‐independent, the subsequent increase in shear stress stimulates NO synthesis by endothelial cells, thereby promoting smooth muscle relaxation and arterial dilatation (endothelium‐dependent vasodilation) [18, 19, 20]. Arterial diameters at rest and at end‐diastole are measured by a digital edge detection software [14]. FMD is calculated as the percentage increase in arterial diameter from baseline to peak dilation [14].

FMD has received considerable attention due to its correlation with coronary artery endothelial dysfunction and the severity of coronary atherosclerosis [18]. Additionally, emerging evidence has shown that low FMD holds prognostic significance for future cardiovascular events in both healthy individuals and those with established cardiovascular disease, such as patients with coronary artery disease, heart failure and hypertension [18, 19].

To date, few studies have investigated macrovascular endothelial function using FMD in patients with single ventricles, with most of them reporting lower FMD values compared to healthy controls [7, 9, 21, 22, 23] (Table 1). The absence of significant differences in the study by Sarkola et al. could be explained by the differences in Fontan surgical strategies, the young age of the study population, limited sample size and methodological factors, including the potential influence of preceding blood pressure measurements on FMD assessment [23]. Interestingly, FMD has been found to inversely correlate with serum symmetric dimethylarginine (SDMA), a potent eNOS inhibitor, in 23 paediatric Fontan patients [9]. In another study, prolonged exposure to hypoxia before Fontan completion has been correlated to diminished FMD, implying that preoperative oxygen deprivation may be implicated in endothelial damage in this population [22]. Moreover, the detected association between FMD and peak oxygen consumption, exercise hyperemic reaction and post‐exercise oxygen resaturation suggests that exercise intolerance in Fontan circulation may be partly associated with abnormal skeletal muscle hemodynamics, which are linked to endothelial dysfunction [7].

**TABLE 1:** Studies evaluating peripheral endothelial dysfunction with functional methods in patients with single ventricle physiology.

Study, publication year	Study type	Method of assessment	Measurement	Study population (*N*)	Patient age (years)	Main findings	Significant associations in SV patients
Mahle et al. 2003 [9]	Case–control	FMD	FMD%	23 Fontan patients (13 with prior aortic arch surgery, 10 without prior aortic arch surgery) and 19 controls	11.8 ± 4.2 years	Lower FMD% in Fontan vs. controls Ns differences in FMD% based on prior aortic arch surgery	FMD% not associated with ventricular morphology, serum L‐arginine or the L‐arginine/ADMA ratio FMD% inversely correlated with serum SDMA Trend for inverse correlation between ADMA and FMD%
Inai et al. 2004 [7]	Case–control	NIRS on the vastus lateralis muscle at rest, during exercise (CPET) and during recovery, FMD and NMD of the brachial and posterior tibial arteries	Changes in HHb, HbO_2_, HbT, SmO_2_ (%) on the vastus lateralis muscle at rest, during exercise (CPET) and during recovery, FMD%, NMD%	50 Fontan and 15 healthy controls	21 ± 7 years	Diminished HbT increase during exercise, muscle oxygen extraction and post‐exercise oxygen resaturation in Fontan vs. controls Diminished FMD% in the brachial and posterior tibial arteries in Fontan patients vs. controls Similar NMD% in both groups in brachial and posterior tibial arteries	HbT increase during exercise, muscle oxygen extraction and post‐exercise oxygen resaturation correlated positively with peakVO_2_ FMD% correlated positively with peakVO_2_, the increase in HbT during exercise and post‐exercise oxygen resaturation
Jin et al. 2007 [22]	Retrospective case–control	FMD and NMD	FMD%, NMD%	44 Fontan patients vs. 25 healthy controls	15 ± 6.1 years	Lower FMD and NMD in Fontan vs. controls	FMD not correlated with IMT FMD inversely correlated with duration of exposure to chronic hypoxia Trend for higher FMD and NMD in patients receiving ACEi compared with those not receiving ACEi
Mahle et al. 2009 [24]	4‐week trial	FMD	FMD% before and after 4 weeks of spironolactone administration	10 Fontan patients	28 (range 15–42) years	FMD did not change significantly after 4 weeks of spironolactone	FMD% at baseline not associated with age and previous history of aortic arch anomaly
Natarajan et al. 2009 [21]	Pilot	FMD	FMD%	18 SV patients (before the Fontan procedure with hypoxemia) and 19 controls	28 ± 25 months	Lower FMD in SV patients vs. controls Ns differences in FMD between pre‐Glenn patients and Glenn patients, between patients receiving ACEi and those who were not, and between patients who had undergone arch reconstruction vs. those who had not	NA
Rao et al. 2009 [25]	Pilot	Four‐site NIRS (brain, kidney, deltoid and vastus lateralis)	rSO2 on the midline forehead (cerebral), below the 12th rib in the left paravertebral space (renal), on the vastus lateralis (leg), and on the deltoid muscle (arm) at rest, during exercise, and throughout a 5‐min recovery period	5 Fontan and 33 controls		Higher initial slope of increasing AVDO_2_ in the cerebral circulation in Fontan patients vs. controls After ventilatory anaerobic threshold, flat AVDO_2_ slope in Fontan patients Larger rebound of cerebral rSO2 after quitting time in Fontan vs. controls	NA
Goldstein et al. 2011 [6]	Prospective case–control	PAT	Baseline pulse amplitude, Endo‐PAT index, PAT ratio	51 asymptomatic Fontan patients and 22 healthy controls	15.0 (10.9–17.8) years	Higher baseline pulse amplitude in Fontan patients vs. controls Lower PAT ratio in Fontan patients vs. controls	Baseline pulse amplitude inversely correlated with PAT ratio Baseline pulse amplitude positively correlated with BMI and self‐reported total, physical, and psychosocial quality of life scores Tobacco exposure associated with a significantly increased baseline pulse amplitude
Danduran et al. 2012 [26]	Case–control	Four‐site NIRS (brain, kidney, deltoid and vastus lateralis)	rSO_2_ on forehead (brain), para vertebral space (kidney), vastus lateralis (leg), and deltoid muscle (arm) at rest, exercise, and throughout a 5‐min recovery period	50 Fontan patients and 50 controls	11.25 (7–23) years	Significant cerebral hyperemia post exercise in Fontan vs. controls (in whom redistribution of flow was directed to somatic muscles) Higher change in rSO_2_ (on brain and leg) from rest to 2 and 5 min after exercise in Fontan vs. controls	NA
Sarkola et al. 2013 [23]	Prospective case–control and cross‐sectinal	FMD	FMD%	28 Fontan patients and 54 controls	14.8 ± 1.3 years	Ns differences in FMD, between Fontan and controls	Ns association between right carotid to right radial PWV or FMD and the use ACEi, anticoagulation, resting oxygen saturation, peak VO_2_ or percent of predicted peak VO_2_, or duration after Fontan completion
Lambert et al. 2013 [8]	Case–control	PAT	RHI, PAT ratio	18 Fontan patients vs. 23 controls	25 ± 1 years	Decreased RHI and PAT ratio in Fontan vs. controls	NA
Loomba et al. 2013 [27]	Retrospective case–control	Four‐site NIRS (brain, kidney, deltoid and vastus lateralis)	rSO_2_ in the forehead midline (brain), below the 12th rib in the left paravertebral space (kidney), on the vastus lateralis (leg) and on the deltoid muscle (arm) during rest, exercise, and a 5‐min recovery period	50 Fontan patients and 51 controls	11.6 ± 3.8	Lower resting renal rSO2 and resting cerebral rSO2 in Fontan vs. controls Lower mean resting cerebral rSO2 and resting renal rSO2 in both unfenestrated and fenestrated Fontan patients vs. controls Greater change from baseline in 2 min into the recovery period in renal rSO_2_ in Fontan patients vs. controls Ns difference in renal rSO2 at peak exercise or 5 min into recovery between Fontan and controls Ns difference in cerebral rSO_2_ change, renal AVDO_2_ and cerebral AVDO_2_ at the time of peak exercise, at 2 min into the recovery, or at 5 min into the recovery between Fontan and controls Ns difference in rSO2 change and AVDO2 in Fontan patients with fenestration vs. those without fenestration	NA
Bertolizio et al. 2015 [28]	Prospective, observational cohort	Cerebral NIRS	Cerebral rSO_2_ (on the right and left forehead) before, during and after the bidirectional Glenn operation	24 infants with SV and surgically placed systemic‐to‐pulmonary artery shunts undergoing the bidirectional Glenn operation	5.6 (3.0–8.2) months	Cerebral rSO2 increased after the bidirectional Glenn operation	NA
Huang et al. 2015 [29]	Cross‐sectional	NIRS of the brain and mesenteric circulation every hour	Cerebral rSO_2_ (on the forehead) and mesenteric rSO_2_ (around the umbilicus) 30 min before, 30 min after and 12 h after extubation	24 patients undergoing bidirectional cavopulmonary shunt	15.93 ± 12.3 months	Cerebral rSO2 increased 30 min and 12 h after extubation Mesenteric rSO2 remained unchanged at 30 min after extubation, but increased 12 h after extubation	Cerebral rSO2 inversely correlated with central venous pressure
Sabri et al. 2016 [30]	Trial	FMD	FMD% before and after 6‐week administration of tadalafil	15 Fontan patients	15.03 ± 8.33 years	Tadalafil had no significant effect on FMD%	NA
Hoffman et al. 2017 [31]	Post hoc analysis of a prospective cohort	Cerebral and somatic NIRS	Cerebral and somatic/renal rSO_2_	194 neonates with HLHS after Stage 1 palliation	8.8 ± 7.3 days	Significantly higher arteriocerebral and somatocerebral NIRS saturation differences for survivors vs. non‐survivors	Cerebral and somatic rSO2 independent predictors of early postoperative survival
Rescoe et al. 2017 [32]	Retrospective cross sectional	Cerebral NIRS	Cerebral rSO_2_ (on the forehead)	71 HLHS neonates after Stage 1 palliation	Neonates	Cerebral rSO_2_ detected ScvO_2_ less than 30% in the range of clinical interest (rSO_2_ 30–50) with low sensitivity, which was improved by correcting rSO_2_ for arterial contamination (by back‐calculating ScvO_2_ based on rSO_2_)	NA
Turquetto et al. 2018 [17]	Cross‐sectional	VOP	FBF (mL/min/100 mL), FVC (units)	30 Fontan patients and 27 controls	18 (15–24) years	Blunted FBF and FVC in Fontan vs. controls	FVC positively correlated with the cross‐sectional area of the thigh and negatively correlated with MSNA
Sandberg et al. 2019 [33]	Case–control and cross‐sectional	NIRS at rest, during isotonic shoulder flexions (0°–90°) to exhaustion, and during recovery	HbO_2_ (μmol), HHb (μmol) and TOI (%) before, during and after exercise Muscle oxygen saturation on the deltoid muscle at rest (%), desaturation rate at exercise onset (%StO_2_x3.5 s^−1^), resaturation rate post exercise (%StO_2_x3.5 s^−1^), fractional oxygen extraction (%), Half recovery time to peak hyperemia post exercise (s)	65 patients with complex CHD (22 Fontan) and 71 controls	35.6 ± 14.3 years	Lower muscle oxygen saturation at rest, slower desaturation rate at exercise onset and slower resaturation rate post exercise in Fontan vs. controls	NA
Agarwal et al. 2019 [34]	Pilot case–control study	Vascular optical spectro‐photometry	Superficial and deep StO_2_	8 Fontan, 9 ToF and 8 with liver disease patients vs. 22 controls	34.5 (23.1–45.8) years	Reduced superficial StO2 in Fontan patients vs. ToF and controls, but not vs. the liver group Deeper StO2 was similar between groups Raised gap between deeper and superficial StO_2_ in Fontan vs. ToF and controls, but not vs. the liver group	NA
Vandekerckhove et al. 2019 [35]	Case control and cross‐sectional	NIRS during incremental ramp exercise test	Changes in the cerebral and muscular HbO_2_ (μmol), HHb (μmol) and TOI (%)	18 Fontan and 20 controls	11.8 ± 2.8 years	Faster and steeper course up to the same level of cerebral HHb and absent increase in cerebral HbO_2_ in Fontans Steady decrease in cerebral TOI from the start of exercise in Fontans Similar muscular HHb curve between patients and controls, with an early cessation. HbO_2_ has a similar pattern, with early discontinuation at a higher HbO_2_‐level Muscular TOI has the same course throughout exercise, starting from a lower level	Amplitude of change in cerebral TOI and muscle TOI negatively correlated with peakVO_2_ and predVO_2_peak
Schroer et al. 2019 [36]	Prospective cohort	Four‐site NIRS during cycle ergometer exercise and daily activities	rSO_2_ on frontal cortex (cerebral), triceps brachii muscle (skeletal muscle‐arm), kidney (renal), quadriceps femoris muscle (skeletal muscle‐leg)	7 Fontan patients with PLE, 22 without PLE and 9 patients with d‐TGA (after arterial switch)	Fontan with PLE: 17.3 ± 6.7, Fontan without PLE: 16.4 ± 5.7 and d‐TGA: 16.2 ± 5.3 years	Ns differences in cerebral and triceps brachii rSO_2_ at baseline, during exercise, recovery and daily activity phase between all groups Reduced renal rSO2 at baseline, exercise and daily activities in Fontan patients with PLE vs. Fontan without PLE and d‐TGA Reduced rSO2 in quadriceps femoris muscle in Fontan vs. d‐TGA patients at all time points Renal threshold analysis: significantly reduced renal rSO2 (rSO2 < 65%) in Fontan patients with PLE during exercise (95% of monitoring time below threshold) and daily activities (83.7% time below threshold)	NA
Stoeker et al. 2019 [37]	Prospective case–control	NIRS	Respiratory Muscle Oxygenation (changes in HHb, HbO_2_, HbT) on the right side of the upper body above the 6th intercostal space at the anterior axillary line over the serratus anterior muscle during CPET	22 male Fontan patients and 10 male controls	12.04 ± 2.51 years	Earlier onset of respiratory muscle deoxygenation in Fontan vs. controls Worse matching of local muscle perfusion to oxygen demand between 50% and 90% peak VO_2_ in Fontan vs. controls	NA
Manning et al. 2021 [38]	Prospective case–control	PAT	RHI PAT‐derived AIx	31 Fontan patients (10 with protein‐losing enteropathy)	14.3 (12.2–18.9) years	Higher PAT‐derived AIx in Fontan vs. controls Lower RHI in Fontan patients vs. controls In patients with protein‐losing enteropathy vs. those without protein‐losing enteropathy: Elevated AI Ns difference in RHI	AIx correlated with cEPC
Bergdahl et al. 2022 [39]	Prospective cross‐sectional	NIRS in gastrocnemius muscle	Changes in HbO2, HHb, HBT and TOI in the medial portion of the gastrocnemius muscle during and after heel raise exercise to exhaustion	43 Fontan patients and 43 controls	12.2 ± 3.9 years	During exercise: lower increase in HHb and slower rate of change in HBT in patients vs. controls Following exercise: slower initial increase in TOI and longer half‐time to maximum hyperemia in patients vs. controls	NA
Gumm et al. 2023 [40]	Prospective Pilot	Four‐site NIRS during rest, exercise (CPET), and a 5‐min recovery period	rSO_2_ on the forehead midline (cerebral), below the 12th rib in the left paravertebral space (renal), over the right upper abdomen (hepatic), and on the vastus lateralis muscle (peripheral muscle) during rest, exercise (CPET), and a 5‐min recovery period	10 Fontan patients	22.0 (19.3–25.5) years	Greatest decrease observed in hepatic and renal rSO2 during exercise The hepatic rSO2 showed the slowest recovery vs. renal, cerebral, and peripheral muscle rSO2	NA
Odanaka et al. 2023 [41]	Retrospective case–control	PAT	RHI	48 patients with repaired CHD (18 Fontan patients) and 114 controls	15.0 ± 3.1	Lower RHI in Fontan vs. controls Ns difference in RHI among repaired non‐cyanotic CHD, repaired ToF and Fontan patients	NA
Tekerlek et al. 2024 [42]	Prospective case–control	NIRS before, during and after CPET	SmO_2_ (%) over the vastus lateralis muscle before, during and after CPET, deoxygenation, reoxygenation	31 Fontan patients and 30 controls	18 (9) years	Lower resting SmO2, minimum SmO2 during test, and recovery SmO2 vs. controls Similar muscle deoxygenation and reoxygenation between groups	NA

Abbreviations: ACEi, angiotensin‐converting enzyme inhibitor; ADMA, asymmetric dimethylarginine; AVDO_2_, arterial–venous oxygen saturation differences; BMI, body mass index; cEPCs, circulating endothelial progenitor cells; FMD, flow‐mediated dilatation; FVC, forearm vascular conductance; HbO_2_, oxygenated haemoglobin concentration; HbT, total haemoglobin concentration; HHb, deoxygenated haemoglobin concentration; HLHS, hypoplastic left heart syndrome; MSNA, muscle sympathetic nerve activity; NA, non‐applicable; NIRS, near‐infrared spectroscopy; NMD, nitroglycerine‐mediated dilatation; PAT, peripheral arterial tonometry; peakVO2, peak oxygen consumption; PLE, protein‐losing enteropathy; PP, pulse pressure; PWV, pulse wave velocity; RAC, relative‐area change; RHI, Reactive Hyperemia Index; rSO2, regional oxygen saturation; ScvO_2_, cerebral venous oxyhemoglobin saturation; SDMA, symmetric dimethylarginine; SmO_2_, skeletal muscle O2; StO2, tissue oxygen saturation; SV, single ventricle; ToF, Tetralogy of Fallot; TOI, tissue oxygenation index; VOP, venous occlusion plethysmography.

The effect of Renin–Angiotensin–Aldosterone System inhibitors on endothelial function is a controversial issue. Experimental data have shown that Angiotensin‐Converting Enzyme inhibitors (ACEi) improve endothelial function by upregulating NO synthase (eNOS) protein expression and reducing reactive oxygen species (ROS) production [41, 43]. Moreover, the addition of spironolactone to ACEi therapy has been reported to further increase NO bioavailability [24]. In the Fontan population, a retrospective case–control study involving 44 patients demonstrated a trend toward higher FMD in those receiving ACEi compared to untreated individuals [22]. Conversely, a pilot study of 18 single‐ventricle patients with hypoxemia prior to Fontan completion found no significant difference in FMD between those receiving ACEi and those who were not [21]. Similarly, a small trial of 10 Fontan patients demonstrated no significant change in FMD following 4 weeks of spironolactone administration [24]. Collectively, these data highlight a discordance between the mechanistic rationale supporting RAAS inhibition and the clinical evidence in Fontan patients, and underscore the need for adequately powered, prospective trials before any therapeutic conclusions can be drawn.

While robust evidence supports the role of phosphodiesterase Type 5 inhibitors in reducing pulmonary vascular resistance—thereby improving passive pulmonary blood flow and increasing ventricular preload—their effects on vascular function remain unclear [30]. To date, only one small study has explored the effects of tadalafil in 15 Fontan patients, reporting no significant changes in FMD [30]. Consequently, any potential effects of phosphodiesterase‐5 inhibition on endothelial function should be regarded as hypothesis‐generating and require confirmation in larger prospective studies.

### 2.3 Nitroglycerin‐Mediated Vasodilatation (NMD)

Nitroglycerin‐mediated dilation (NMD) is methodologically similar to FMD, as both techniques assess vascular function through changes in arterial diameter. However, the key difference lies in the underlying mechanism: while FMD evaluates endothelium‐dependent vasodilation in response to shear stress–induced release of endogenous NO, NMD assesses endothelium‐independent vasodilation by directly administering an exogenous NO donor, typically via a sublingual dose of nitroglycerin [20]. This allows for the evaluation of vascular smooth muscle cell responsiveness independently of endothelial function [20]. Consequently, NMD should be performed in conjunction with FMD to confirm that low FMD values are attributed to endothelial dysfunction rather than diminished vascular smooth muscle responsiveness [20].

Studies applying NMD in Fontan patients are scarce and yield conflicting results. An early case–control study involving 50 Fontan patients reported no significant differences in NMD between patients and healthy controls [7]. In contrast, a more recent study of 44 Fontan patients demonstrated impairment in both endothelium‐dependent and endothelium‐independent vasodilation, suggesting that this population may exhibit not only reduced endogenous NO production in response to shear stress but also diminished vascular smooth muscle responsiveness to exogenous NO donors [22].

### 2.4 Near‐Infrared Spectroscopy

More evidence exists on the application of Near‐Infrared Spectroscopy (NIRS) in patients with Fontan circulation. Although not a direct measure of endothelial function, NIRS is a non‐invasive method for evaluating regional tissue oxygenation or perfusion and can obtain measurements of local oxygen consumption, metabolism and blood flow [15]. This method is utilized to investigate hemodynamic responses in cerebral, skeletal muscle tissues, kidneys and other visceral organs as skin blood flow variations are considered to have negligible impact on the recorded measurements [15]. A typical NIRS device includes a light source, emitting light in the near‐infrared range into the tissue of interest, and two detectors for measuring tissue oxygen levels at different depths (one measuring superficial and the other deep tissue oxygenation) [44]. Haemoglobin (Hb) and myoglobin (Mb), oxygen carriers in blood and muscles respectively, absorb light differently depending on their oxygenation [44]. NIRS facilitates monitoring of skeletal muscle oxygenation and microvascular reactivity both at rest and during exercise by capturing functional changes in oxygenated Hb dissociation [16]. More specifically, the difference in light absorption by Hb reflects the oxygen uptake of the examined tissue and is referred to as regional oxygen saturation (rSO2).

Combining NIRS with arterial or venous occlusion enables the measurement of regional skeletal muscle oxygen consumption (VO_2_) [44]. Arterial occlusion establishes a closed system without blood flow, whereby the rate of decline in oxygenated haemoglobin (or the corresponding increase in deoxygenated haemoglobin) reflects muscle VO_2_. In the case of venous occlusion, venous outflow is restricted, and the rate of increase in deoxygenated haemoglobin indicates VO_2_, while the rate of increase in total haemoglobin serves as a measure of resting blood flow [44].

Under normal conditions, muscle blood flow increases markedly during exercise. During rhythmic exercise, the mechanical action of the muscle pump induces volumetric fluctuations within the vasculature that appear as cyclic changes in oxy‐ and deoxyHb signals [44]. Using standardized protocols, this hemodynamic response enables the estimation of VO_2_ during exercise, as well as the post‐exercise recovery of VO_2_—an established indicator of skeletal muscle oxidative capacity [44]. Given that reoxygenation is impaired in individuals with compromised oxygen delivery, NIRS may serve as a valuable tool for evaluating muscle oxygenation in patients with Fontan circulation [44].

Indeed, cerebral NIRS has been widely used in congenital heart surgery, providing reliable cerebral perfusion monitoring and playing a well‐studied role in perioperative patient management [28, 29, 31, 32]. In a prospective study of infants with single‐ventricle lesions and surgically placed systemic‐to‐pulmonary artery shunts, cerebral rSO_2_ was found to improve after the bidirectional Glenn operation [28]. Another similar study in children undergoing the Glenn procedure examined the effects of extubation on regional tissue oxygen saturation and demonstrated a significant increase in cerebral rSO_2_ post‐extubation, suggesting that cerebral blood flow may be relatively compromised during positive pressure ventilation [29]. Notably, continuous non‐invasive cerebral NIRS monitoring also possesses prognostic significance, as early postoperative changes in rSO2 within the first 6 h independently predicted survival in a cohort of 194 neonates with hypoplastic left heart syndrome (HLHS) following initial surgical palliation [31].

In addition to cerebral haemodynamics, several studies have investigated muscle oxygenation using NIRS in the Fontan population [7, 25, 26, 27, 33, 35, 36, 37, 39, 40, 42]. A case–control study assessed NIRS‐derived skeletal muscle O_2_ saturation at rest, during isotonic shoulder flexions (0°–90°) to exhaustion, and during recovery, revealing slower oxygenation kinetics in Fontan patients compared to controls [33]. This altered skeletal muscle metabolism was confirmed by subsequent studies [35, 39, 42]. Inai et al. explored skeletal muscle haemodynamics during exercise in 50 Fontan patients and 15 controls, demonstrating attenuated exercise hyperemic reaction and post‐exercise oxygen resaturation in Fontan circulation [7]. These findings correlated with peak oxygen consumption, suggesting that exercise intolerance in this population may, in part, result from impaired skeletal muscle hemodynamics [7]. Moreover, the demonstrated association between FMD and both the exercise‐induced hyperemic response and post‐exercise oxygen resaturation in this study underscores a potential contribution of endothelium‐dependent vasodilation to the regulation of blood flow in the working skeletal muscle [7].

Four‐site NIRS (brain, kidney, deltoid, vastus lateralis) has also been employed in Fontan circulation during ramping exercise to investigate potential differences between patients with and without fenestration [27]. Comparisons between these two groups revealed no statistically significant differences in exercise duration, changes in cerebral or muscle rSO_2_, or arterial–venous oxygen saturation differences (AVDO_2_), suggesting that fenestration closure does not significantly affect the dynamics of regional oxygen extraction during either exercise or recovery [27].

Renal NIRS may also be valuable; a small cohort demonstrated lower renal rSO_2_ in Fontan patients with protein‐losing enteropathy (PLE) compared to those without, with renal desaturation disproportionately exceeding the concomitant reduction in peripheral SpO_2_ [36]. This implies that renal rSO_2_ may serve as a more sensitive indicator of tissue oxygenation, irrespective of ventricular function and peripheral SpO_2_ [36]. Given that persistently reduced rSO_2_ levels in Fontan patients with PLE may indicate a risk of chronic kidney disease driven by recurrent renal hypoxemia and ischemic injury, renal NIRS monitoring could be a valuable non‐invasive tool for early detection of renal dysfunction and may even outperform pulse oximetry in identifying patients at risk [36]. Furthermore, decreased renal rSO_2_ may reflect broader splanchnic hypoxemia due to elevated venous and lymphatic pressures, resulting in microcirculatory disturbances and endothelial injury that may contribute to the pathophysiology of PLE [36].

### 2.5 Peripheral Arterial Tonometry

Peripheral arterial tonometry (PAT) is a user‐friendly technique that noninvasively assesses digital microvascular reactivity through the measurement of pulsatile arterial volume changes using finger plethysmography in an observer‐independent manner [18, 45]. The PAT device consists of two finger‐mounted probes designed to detect pulsatile volume changes in the vessels of the index finger of each hand (test and control). The control finger provides reference data to account for systemic physiological fluctuations during the procedure [46]. The examination probe features a stiff external covering containing the electronically controlled inflatable chambers. These chambers apply a standardized pressure of 70 mmHg on the index finger, effectively preventing venous pooling and blood stasis, thereby minimizing the risk of triggering reflex vasoconstrictive responses [18, 46]. A blood pressure cuff is positioned on the study arm, while the contralateral arm acts as an internal control enabling correction for any potential systemic drift in vascular tone during the procedure [14, 18].

The PAT test is conducted in three consecutive phases—baseline, occlusion and reactive hyperemia—each lasting approximately 5 min [46]. Following an initial 5 min baseline recording, the blood pressure cuff is inflated to suprasystolic pressure for 5 min to induce transient ischemia [18, 45, 46]. Upon cuff release, a reactive hyperemic response is elicited and recorded for an additional five minutes [18, 45, 46]. In healthy individuals, this increase in arterial blood volume leads to an increased pulsatile volume in the fingertip, enhancing the recorded signal [18, 46]. In contrast, a blunted response is indicative of endothelial dysfunction [18, 46]. Post‐deflation amplitude changes detected in the control finger are attributed to systemic effects [46].

The ratio between hyperemic and baseline pulse volume amplitude (PAT ratio) is normalized to the control arm (using a baseline correction factor), yielding the Reactive Hyperemia Index (RHI) [45, 46]. After cuff deflation, the RHI is calculated by the software at 30‐s intervals, with peak hyperemic response at 90–120 s [46]. Values under 1.67–2.0 are commonly used as the threshold for impaired endothelial function, whereas higher values typically reflect normal vascular function [46]. Additional validated metrics derived from PAT include the log‐transformed PAT‐RHI and the Framingham Heart Study–modified RHI [46]. Beyond peripheral reactive hyperemia data, the Augmentation Index (AIx) can also be derived from pulse waveform analysis of the PAT signal [14, 46]. The systolic peak (P1) and the reflected wave peak (P2) are automatically identified and used to calculate the AIx with the formula: AIx = ((P2 − P1)/P1) × 100 [14, 46].

PAT holds considerable promise for advancing cardiovascular research as reduced PAT measurements have been shown to correlate with coronary microvascular impairment and predict adverse cardiovascular events [18]. Despite numerous studies reporting inconsistent findings on the relationship between FMD and PAT, two large population‐based studies that employed both methods identified only a modest association, suggesting that FMD and PAT may reflect distinct aspects of vascular function [18].

Evidence from studies in Fontan circulation has consistently demonstrated reduced PAT ratio and RHI in patients compared to controls, indicating impaired peripheral digital vasodilatory function even in asymptomatic patients [6, 8, 38, 41]. In a prospective case–control study including 51 Fontan patients and 22 controls, baseline digital pulse amplitude was found significantly elevated in the Fontan group [6]. This finding could possibly be explained by the decreased digital microvascular tone, increased pulse pressure, increased blood flow and structural alterations in the microvasculature inherent to the Fontan physiology [6]. Notably, patients within the Fontan group with a history of tobacco use or higher body mass index exhibited even greater baseline pulse amplitudes, indicative of increased brachial blood flow at rest and potential vessel damage [6]. Consequently, the combined effects of Fontan physiology and tobacco exposure or obesity may put this population at a heightened risk of endothelial dysfunction [6].

In a study of 31 adolescents with Fontan circulation, PAT‐derived AIx was significantly higher compared to controls, indicating increased arterial stiffness following the Fontan operation [38]. Interestingly, AIx was significantly elevated in patients with protein‐losing enteropathy than in those without protein‐losing enteropathy [38]. This finding reinforces the concept that end‐organ complications, such as protein‐losing enteropathy, further exacerbate endothelial dysfunction through multiple pathways that contribute to a pro‐inflammatory and hypoxic state, perpetuating a vicious cycle of vascular damage [10].

## 3 Assessment of Arterial Stiffness in Fontan Circulation

The vascular endothelium is a key regulator of vascular tone and, as a result, a major determinant of arterial mechanical properties, including arterial stiffness and its inverse, arterial elasticity [47]. Increased arterial stiffness augments the amplitude of the forward pressure wave generated by the ventricle, and causes earlier systolic arrival of the reflected waves, which further elevate the aortic systolic pressure, increase systolic ventricular afterload, and reduce diastolic coronary perfusion pressure [48]. Consequently, arterial stiffness is widely recognized as an important biomarker of cardiovascular disease and is closely associated with the severity and extent of coronary artery disease [48]. Although direct assessment of central arterial stiffness is challenging, it can be reliably evaluated using validated surrogate markers, for example, pulse wave velocity (PWV), arterial distensibility, the β‐stiffness index, characteristic impedance (Zc) and AIx.

### 3.1 Pulse Wave Velocity

PWV is widely regarded as the ‘gold‐standard’ method for assessing directly arterial stiffness. Theoretically, PWV can be calculated using the Moens and Korteweg formula: PWV^2^ = Eh/2rρ [49], according to which, PWV depends on the geometry of the artery (thickness, *h*; and radius, *r*), the intrinsic elastic properties of the arterial wall (elastic incremental modulus, *E*) and the blood density (*ρ*) [49]. In clinical practice, PWV is determined as the ratio of the distance between two arterial recording sites (Δ*L*) to the time required for the pulse wave to propagate between them (Δ*T*), with measurements obtained either simultaneously at both measurement sites or from sequential recordings temporally aligned to a fixed reference point in the cardiac cycle, typically the R wave of the electrocardiogram [48, 49].

Apart from infrequently employed invasive pressure catheter techniques, non‐invasive methods have been used to assess aortic stiffness. These include local measurements, for example, magnetic resonance imaging (MRI) and ultrasound systems, regional measurements for example, transit time of pressure waveform from probes or cuffs (applanation tonometry, photoplethysmography, oscillometry), and indirect measurements derived from pressure waveforms from a single brachial cuff pressure recording [49, 50].

PWV is measured along the aortic and aorto‐iliac pathways and may also be assessed in peripheral arteries; however, aortic PWV is considered the most clinically relevant [51]. The most commonly used proxy for aortic PWV is carotid–femoral PWV, in which pulse transit time is derived from recordings obtained at the carotid and femoral arteries [49], which are anatomically proximal to the aorta, allowing it to reliably reflect central arterial stiffness and be regarded as the gold standard [49]. PWV can also be assessed in other segments of the arterial tree, including the brachial–tibial arteries (brachial–ankle PWV, baPWV), the heart–tibial arterial pathway (cardio‐ankle vascular index, CAVI), the carotid–radial (carotid‐radial PWV) and radial–femoral arterial segments (radial‐femoral PWV) [48].

In functionally univentricular hearts, increased PWV is of particular significance, as even small elevations in afterload may precipitate ventricular dysfunction, a major contributor to Fontan circulation failure [52]. PWV has been evaluated in Fontan patients using several non‐invasive techniques, yielding conflicting findings (Table 2). Prospective studies employing applanation tonometry have reported increased carotid artery PWV and carotid‐radial PWV in Fontan patients compared to controls [23]. An additional study using MRI demonstrated increased ascending aortic PWV in children with HLHS, while oscillometric methods have also reported higher aortic PWV in Fontan patients compared with controls [61, 63]. In contrast, studies utilizing Doppler ultrasound or applanation tonometry have found no significant differences in carotid–femoral PWV between single‐ventricle patients and controls [8, 21, 23, 57], and other MRI‐based studies have similarly reported no differences in aortic PWV [54, 61]. These discrepancies may be attributed to the differences in methodology, the aortic segment assessed and patient characteristics across studies. Several studies included relatively young Fontan patients, in whom vascular abnormalities may not yet be fully established, given that vascular dysfunction appears to evolve progressively over time. Additionally, many studies were limited by relatively small sample sizes, potentially reducing their statistical power to detect modest but clinically relevant differences in arterial stiffness.

**TABLE 2:** Studies evaluating arterial stiffness with non‐invasive techniques in patients with single ventricle physiology.

Study, publication year	Study type	Method of assessment	Measurement	Study population (*N*)	Patient age (years)	Main findings	Significant associations in SV patients
Cardis et al. 2006 [53]	Prospective cross‐sectional	Transoesophageal echocardiography	Distensibility index, stiffness index (β)	20 HLHS patients (with aortic arch reconstruction and bidirectional Glenn/Fontan), 18 SV patients without arch obstruction or hypoplasia and 22 patients with double‐ ventricular lesions without arch obstruction or hypoplasia	22.2 months	In HLHS patients vs. patients with non‐HLHS SV lesions or two‐ventricle lesions: lower distensibility index higher stiffness index	NA
Natarajan et al. 2009 [21]	Pilot	Doppler ultrasound	Carotid‐femoral PWV (m/s)	18 SV patients (before the Fontan procedure with hypoxemia) and 19 controls	28 ± 25 months	Ns differences in PWV between SV patients and controls Ns difference in PWV between patients who had undergone arch reconstruction vs. those who had not	NA
Voges et al. 2010 [54]	Case–control and cross‐sectional	MRI	Distensibility in the AAo, aortic arch and DAo (10^−3^ mmHg^−1^), aortic PWV (m/s)	40 patients with HLHS (undergone 3‐stage surgical palliation with reconstruction of the aortic arch) and 13 controls	6.0 ± 2.2 years	Decreased distensibility at the AAo and the transverse aortic arch in HLHS vs. controls Ns difference in PWV and DAo distensibility between patients and controls	AAo distensibility negatively correlated with the EF and the amount of late gadolinium enhancement
Sarkola et al. 2013 [23]	Prospective case–control and cross‐sectional	Applanation tonometry	Abdominal aortic and carotid artery stiffness indices, carotid‐femoral and carotid‐radial PWV (m/s)	28 Fontan patients and 54 controls	14.8 ± 1.3 years	Ns differences in abdominal aortic and carotid artery stiffness indices and carotid‐femoral PWV between Fontan and controls Higher carotid‐radial PWV in Fontans vs. controls	Ns association between right carotid to right radial PWV or FMD and the use ACEi, anticoagulation, resting oxygen saturation, peak VO_2_ or percent of predicted peak VO_2_, or duration after Fontan completion
Lambert et al. 2013 [8]	Case–control	Applanation tonometry	Carotid‐femoral PWV (m/s), AIx	18 Fontan patients vs. 23 controls	25 ± 1 years	Increased digital and central AIx in Fontan vs. controls Ns differences in cfPWV between the groups	Patients with reduced ventricular function (LVEF < 55%) had increased PWV
Myers et al. 2013 [55]	Prospective case–control study	Carotid artery applanation tonometry	PWV (cm/s), elastic pressure‐strain modulus (Ep, torr), stiffness index (*β*), input impedance (Zi, dyne×sec/cm^5^/m^2^), characteristic impedance (Zc, dyne×sec/cm^5^/m^2^), total arterial compliance (TAC, mL/Torr/m^2^)	22 Fontan patients and 31 healthy controls	14.9 (7.6–18.7) years	Increased PWV, Ep, and b indexes and lower Zi in Fontan patients vs. controls Ns differences in TAC and Zc between patients and controls	NA
Fogel et al. 2014 [56]	Retrospective	CMR	DAo distensibility (10^–3^ mmHg^−1^), aortic PWV (m/s)	126 SV patients (23 prior to bidirectional Glenn, 45 with bidirectional Glenn, 58 with Fontan), 75 with aortic reconstruction and 51 without aortic reconstruction	8.6 ± 8.0 years	Higher PWV in the reconstruction group vs. the no‐reconstruction group In reconstruction group: higher PWV in patients > 13 years vs. those < 7 years old Ns differences in DAo distensibility between groups	PWV not correlated with blood pressure, heart rate, ventricular volumes, ventricular mass, EF or cardiac index
Tomkiewicz‐Pajak et al. 2014 [57]	Case–control	Applanation tonometry	Carotid femoral PWV (m/s), AP, AIx, brachial and central PP (mmHg)	25 Fontan patients vs. 25 controls	24.7 ± 6.2 years	Elevated AP and AIx in Fontan vs. controls Ns differences in carotid‐femoral PWV, brachial and central PP between the groups	PWV negatively correlated with SatO_2_ and positively correlated with WBC, INR, TNFα and postoperative time AIx positively correlated with age at surgery Bilirubin level positively correlated with brachial PP and central PP SatO_2_ independent predictor of PWV
Bhat et al. 2015 [58]	Prospective case–control	Applanation tonometry	AIx, AP, PP (mmHg)	22 Fontan patients vs. 22 controls	13.1 ± 4.2 years	AIx significantly higher in Fontan patients vs. controls	Ns difference in AIx between patients who had undergone arch repair and those who had not Ns difference in AIx based on the SV morphology
Müller et al. 2015 [59]	Prospective case–control and cross‐sectional	Oscillometry	AIx	1125 CHD patients (87 Fontan) and 322 controls	23.9 ± 10.5 years	Fontan circulation independent predictor of AIx	In the whole cohort of CHD: AIx negatively associated with peak VO_2_ and oxygen saturation at rest
Voges et al. 2015 [60]	Case–control and cross‐sectional	MRI	distensibility in the AAo, aortic arch and DAo (10^−3^ mmHg^−1^), aortic PWV (m/s), impedance (Pa s cm^−3^)	79 patients with HLHS (undergone Norwood operation) and 18 controls	6.3 ± 3.2 years	Higher PWV in the aortic arch and the thoracic DAo in HLHS patients with increased CSA of the DAo vs. HLHS patients with normal CSA of the DAo Ns difference in the DAo distensibility between patients and controls Reduced AAo and aortic arch distensibility in patients vs. controls Lower DAo impedance in HLHS patients with dilated DAo vs. those without dilation	Aortic arch PWV correlated with the CSA of the DAo
Schaefer et al. 2019 [61]	Prospective case–control	MRI	AAo and DAo PWV (m/s), AAo and DAo RAC (%)	37 Fontan (19 HLHS and 18 single left ventricle) and 18 controls	11.2 ± 5.5 years	Elevated AAo PWV and reduced RAC in HLHS vs. controls Ns difference in AAo PWV but reduced AAo RAC in patients with single left ventricle vs. controls Ns differences in DAo PWV and DAo RAC between all groups	AAo PWV positively correlated with EDVi and ESVi and negatively correlated with EF and ventricular‐vascular coupling ratio AAo RAC negatively correlated with EDVi, ESVi and z score of ascending aortic size and positively correlated with EF and ventricular‐vascular coupling ratio
Biko et al. 2019 [62]	Prospective cross‐sectional	real‐time exercise CMR	PWV and distensibility of the DAo	48 Fontan patients (18 after aortic reconstruction and 30 without aortic reconstruction)	Reconstructed group: 17.4 (range: 12–24) years, non‐reconstructed group: 17.0 (range: 13–26) years	Higher PWV in the reconstructed group vs. the non‐reconstructed group Ns difference in DAo distensibility between the reconstructed and non‐reconstructed aortic groups	In the reconstructed group: PWV inversely correlated with EDVi, ESVi and SVi at rest and at exercise DAo distensibility inversely correlated with peak oxygen pulse, peak VO_2_, oxygen consumption at ventilatory anaerobic threshold and peak work
Noortman et al. 2019 [63]	Case–control and cross sectional	Oscillometry	Aortic PWV (m/s), AIx	17 Fontan patients and 26 controls	19.12 ± 9.1	Higher PWV and AIx in Fontan vs. controls	PWV and AIx did not correlate with peak VO_2_, BMI and sport participation
Harteveld et al. 2022 [64]	combination of cross‐sectional and prospective intervention study	Oscillometry	aortic PWV (m/s) and AIx, during supine rest and during head‐up tilt testing	35 Fontan patients and 34 controls	14.0 (12.6–16.7) years	Increased aortic PWV and AIx in the supine position in Fontan vs. controls	NA

Abbreviations: AAo, ascending aorta; ACEi, angiotensin‐converting enzyme inhibitor; ADMA, asymmetric dimethylarginine; AIx, Augmentation Index; AP, augmentation pressure; BMI, body mass index; BNP, brain natriuretic peptide; CSA, cross‐sectional area; DAo, descending aorta; EDVi, end‐diastolic volume index; ESVi, end‐systolic volume index; HLHS, hypoplastic left heart syndrome; NA, non‐applicable; peakVO2, peak oxygen consumption; PP, pulse pressure; PWV, pulse wave velocity; RAC, relative‐area change; rSO2, regional oxygen saturation; SV, single ventricle; SVi, stroke volume index.

Most studies have not stratified patients by single‐ventricle morphology; nevertheless, available evidence indicates that individuals who have undergone aortic arch reconstruction, most commonly in the context of HLHS, exhibit higher PWV than those without reconstruction, suggesting that arch surgery may be an important contributor to increased arterial stiffness in this population [56, 62]. Furthermore, the positive correlation of TNF‐α and the inverse correlation of oxygen saturation with carotid–femoral PWV indicate that the extent of systemic inflammation and hypoxia may mirror the severity of vascular dysfunction [57]. Finally, the inverse association between PWV and both ejection fraction and aortoventricular coupling confirms the hypothesis that increased ascending aortic stiffness may contribute to higher afterload and compromised ventricular function in the single‐ventricle circulation [58, 61].

### 3.2 Pulse Wave Analysis

Pulse wave analysis (PWA), another method to evaluate artery properties and arterial stiffness, is ideally assessed at central arteries, such as the carotid artery or the ascending aorta, either through direct measurement or by reconstructing central waveforms from radial artery recordings using a validated transfer function [65]. The arterial pressure waveform results from the composite of the forward pressure wave created by ventricular contraction and a reflected wave from the periphery [65]. PWA is typically performed using applanation tonometry or echo‐tracking methods [66] and enables the calculation of wave reflection indices, for example, central systolic pressure, pulse pressure (PP, difference between systolic and diastolic blood pressure), augmentation pressure (AP, the difference between the first and second systolic peaks) and the AIx [65]. AIx reflects the ratio of the augmentation of systolic pressure induced by the reflected waves to the PP and is calculated as the difference between the second and first systolic peaks expressed as a percentage of the PP (AIx = 100 × ((P2 − P1)/PP)) [48]. Given its strong dependence on heart rate, AIx is typically standardized to a heart rate of 75 beats per minute (AIx75) [66].

Data on PWA indices in Fontan patients remain limited. Case–control studies employing applanation tonometry have reported higher AP and AIx in Fontan patients compared to controls [8, 57, 58], while increased AIx has similarly been observed using oscillometric techniques [63]. In contrast to PWV, increased AIx in single‐ventricle patients did not differ significantly according to the presence of aortic arch reconstruction or ventricular morphology (single left or right ventricle) in a prospective study of 25 Fontan patients [58]. Furthermore, the observed positive correlation between AIx and age at surgery may be explained by prolonged exposure to chronic cyanosis and volume overload prior to surgical intervention, both of which are known to contribute to increased arterial stiffness [57].

### 3.3 Other Indices

Beyond PWV and AIx, several additional indices can be used to characterize the aortic elastic properties. Arterial distensibility describes the relative change in cross‐sectional area in response to a given change in arterial pressure between diastole and systole and is normalized to the diastolic luminal area (*A*), yielding the distensibility coefficient (DC = ΔΑ/Α·ΔP) [52]. Characteristic impedance (Zc) determines the associated pressure change for a given flow change and is directly proportional to wave speed (*c*) and inversely proportional to the vessel area (Zc = *ρ*·*c*/*A*) [52]. The β‐stiffness index is a pressure‐independent measure of arterial stiffness that quantifies the relationship between changes in arterial diameter and blood pressure between diastole and systole and is defined as: *β* = ln(SBP/DBP)/((Ds − Dd)/Dd) where Dd, diastolic diameter, and Ds, systolic diameter [52].

Studies in the Fontan population have demonstrated reduced distensibility and increased stiffness index in the ascending aorta and the reconstructed transverse arch in patients with HLHS following Norwood palliation [53, 54, 60], while no significant differences in descending aortic distensibility have been observed [54, 61, 62]. It appears, therefore, that the decrease in elasticity in this population is localized to the reconstructed arch and proximal ascending aorta, and does not extend to the descending aorta, which suggests that these changes are primarily driven by surgical factors—such as the use of noncompliant homograft or patch material—rather than by intrinsic abnormalities of the native aortic tissue [53]. Moreover, a cross‐sectional MRI study of 40 patients with HLHS showed that reduced ascending aortic distensibility correlated with impaired systolic RV function and the extent of aortic late gadolinium enhancement, indicative of fibrosis and scarring, which may contribute to an increased risk of future RV failure [54].

## 4 Clinical Implications and Future Perspectives

Despite accumulating evidence on endothelial dysfunction in Fontan circulation, significant gaps remain regarding the clinical utility of endothelial assessment techniques and their associations with adverse cardiovascular outcomes. Associations identified in observational studies should not be interpreted as evidence of causality. Additionally, interpretation of the available evidence should be undertaken with caution, given the considerable heterogeneity of Fontan cohorts across studies. Differences in age, ventricular morphology, underlying diagnosis, history of aortic arch reconstruction, fenestration status, comorbidity burden and surgical characteristics are likely to influence vascular structure and function and may contribute to the variability observed among studies. Additionally, as population‐ and lesion‐specific normal reference values are currently lacking, longitudinal assessment of serial changes may be more informative than reliance on isolated measurements or absolute values. Nevertheless, owing to their non‐invasive nature and ease of application, these endothelial assessment techniques could facilitate individualized risk stratification in patients with Fontan circulation, thereby improving prognostic precision and enabling more tailored clinical management.

Impaired endothelial function and abnormal tissue oxygenation have been associated with reduced exercise capacity and may contribute to exercise intolerance through impaired skeletal muscle perfusion and oxygen delivery. Similarly, increased arterial stiffness could potentially promote ventricular–arterial uncoupling, augment ventricular afterload, and contribute to progressive ventricular dysfunction. Abnormalities in regional tissue oxygenation and microvascular function could theoretically help identify patients at increased risk for end‐organ complications, including protein‐losing enteropathy, renal dysfunction, and Fontan‐associated liver disease, reflecting the broader impact of Fontan physiology on systemic vascular and organ function (Table 3). However, the incorporation of such methods into prognostic tools for predicting adverse cardiovascular outcomes requires further research and remains currently far from routine clinical practice. Future prospective studies are warranted to clarify the prognostic significance of vascular dysfunction and to determine whether potential therapies targeting the vascular compartment of Fontan circulation may provide additional clinical benefit beyond the treatment of haemodynamic abnormalities.

**TABLE 3:** Summary of vascular functional techniques in Fontan circulation.

Method	Primary vascular domain assessed	Fontan‐related aspect theoretically anticipated
Venous occlusion plethysmography	Endothelium dependent and independent vasodilation	Venous congestion
Flow‐mediated dilatation	Endothelial macrovascular dysfunction (endothelium‐dependent)	Exercise intolerance
Nitroglycerin‐mediated vasodilatation	Vascular smooth muscle responsiveness (endothelium‐independent)	Exercise intolerance
Near‐infrared spectroscopy	Regional tissue oxygenation/perfusion, local oxygen extraction, metabolism and blood flow	End‐organ complications (protein‐losing enteropathy, Fontan‐associated liver disease, renal dysfunction), exercise intolerance
Peripheral arterial tonometry	Microvascular reactivity	End‐organ complications (protein‐losing enteropathy, Fontan‐associated liver disease, renal dysfunction)
Pulse wave velocity	Arterial stiffness	Ventricular diastolic dysfunction/Fontan failure
Pulse wave analysis (Augmentation Index, augmentation pressure)	Arterial stiffness	Ventricular diastolic dysfunction/Fontan failure

## 5 Conclusions

Patients with Fontan circulation exhibit impaired macro‐ and microvascular function together with increased arterial stiffness that can be adequately quantified by several non‐invasive functional methods, supporting the concept that vascular dysfunction represents a measurable systemic phenotype of Fontan physiology. Given the intricate relationship between vascular injury and adverse cardiovascular outcomes, further investigation is required to define the prognostic significance of vascular dysfunction in Fontan circulation. Prospective longitudinal studies should evaluate whether interventions aimed at improving endothelial health can alter the natural history of the disease and mitigate future cardiovascular risk. The development of standardized assessment protocols and physiology‐ and age‐specific reference values will be essential to facilitate the integration of vascular metrics into clinical practice. Finally, combining vascular function assessment with cardiopulmonary exercise testing, multimodality cardiac imaging and clinical outcome measures may provide a more comprehensive characterization of Fontan physiology and help establish the clinical value of vascular biomarkers in this complex patient population.

## Author Contributions

Conceptualization: A.B., G.G.; Supervision: G.G.; Writing – original draft: A.B.; Writing – review and editing: E.P., T.D., V.K., A.Z., G.G.

## Funding

The authors have nothing to report.

## Ethics Statement

The research reported in this paper adhered to relevant ethical guidelines.

## Consent

The authors confirm that patient consent is not applicable to this article.

## Conflicts of Interest

The authors declare no conflicts of interest.

## Data Availability

Data sharing not applicable—no new data generated, or the article describes entirely theoretical research.
